# 8-Week *Kaempferia parviflora* Extract Administration Improves Submaximal Exercise Capacity in Mice by Enhancing Skeletal Muscle Antioxidant Gene Expression and Plasma Antioxidant Capacity

**DOI:** 10.3390/antiox13091147

**Published:** 2024-09-23

**Authors:** Jiapeng Huang, Yishan Tong, Shuo Wang, Takashi Tagawa, Yasuhiro Seki, Sihui Ma, Ziwei Zhang, Tiehan Cao, Haruki Kobori, Katsuhiko Suzuki

**Affiliations:** 1 Graduate School of Sport Sciences, Tokorozawa Campus, Waseda University, Tokorozawa 359-1192, Japan; 2 Research Center, Maruzen Pharmaceuticals Co., Ltd., Fukuyama 729-3102, Japan; 3 Faculty of Human Sciences, Waseda University, Tokorozawa 359-1192, Japan; 4 Faculty of Sport Sciences, Waseda University, Tokorozawa 359-1192, Japan

**Keywords:** *Kaempferia parviflora*, exercise tolerance, oxidative stress, antioxidant, Nrf2, voluntary wheel running

## Abstract

Black ginger (*Kaempferia parviflora*) extract (KPE) is extracted from a ginger family plant grown in Thailand. The polyphenolic components have potential antioxidant effects and have been reported to enhance exercise performance. However, the impact of long-term KPE administration combined with long-term training on the endurance exercise performance of healthy individuals has not been fully studied. In this study, a healthy mouse model was used to investigate the effects of 8 weeks KPE administration and voluntary wheel running on the submaximal endurance exercise capacity and its mechanism. The results showed that 8 weeks of KPE administration significantly enhanced the submaximal endurance exercise capacity of mice and extended the daily voluntary wheel running distance. By measuring oxidative stress markers in plasma and the mRNA expression of antioxidant genes in skeletal muscle, we found that KPE significantly increased plasma antioxidant levels and activated the Nrf2 (Nuclear factor erythroid 2-related factor 2)/ARE (Antioxidant Response Element) pathway and its downstream antioxidant genes expression in skeletal muscle. These results suggest that KPE may enhance the antioxidant capacity of plasma and skeletal muscle by activating the Nrf2-ARE-centered antioxidant pathway, thereby increasing the daily running distance and improving the submaximal endurance exercise capacity of mice.

## 1. Introduction

Aerobic lives rely on the balance between the stable formation of pro-oxidants and the rate of antioxidant consumption [1]. When pro-oxidants are produced in excessive amounts or the rate of antioxidant generation or consumption declines, oxidative damage accumulates, potentially leading to pathophysiological events [2]. Oxidative stress (OS) refers to a state in which the balance between oxidation and antioxidation in the body is disrupted, favoring oxidation [3]. This specifically happens when the generation of reactive oxygen species (ROS) and reactive nitrogen species (RNS) surpasses the body’s antioxidant defenses, leading to damage to biological macromolecules such as DNA, proteins, and lipids [4]. This imbalance can cause cellular and tissue damage and is closely associated with various diseases and the aging process [5].

Reactive oxygen species, including superoxide anion (O_2_^•−^), hydrogen peroxide (H_2_O_2_), hydroxyl radical (^•^OH), and others, along with reactive nitrogen species such as nitric oxide (NO) and peroxynitrite (ONOO^−^) [5], are generated during normal cellular metabolism [6]. Due to the unpaired electrons, they are extremely reactive and can rapidly react with biomolecules such as DNA, proteins, and lipids, causing oxidative damage [7]. In organisms, antioxidants are substances that can significantly delay or prevent the oxidation of other molecules at low concentrations [3]. These antioxidants safeguard cells and tissues from oxidative damage by neutralizing reactive molecules such as free radicals, ROS, and RNS, thereby halting their chain reactions and facilitating the repair of damaged molecules [2]. Antioxidants are categorized into three major types: 1. Antioxidant enzymes such as superoxide dismutase (SOD), catalase (CAT), and glutathione peroxidase (GPx); 2. Non-enzymatic antioxidants including glutathione, vitamins C and E, and beta-carotene; 3. Preventative or repair systems, comprising complex enzymes that mend damaged DNA, proteins, oxidized lipids, and peroxides, such as lipases, proteases, and DNA repair enzymes [8]. In recent years, the role of reactive sulfur species (RSS) in oxidative stress has garnered significant attention. RSS, including hydrogen sulfide (H_2_S) and its derivatives, such as polysulfides, can directly react with reactive oxygen species (ROS) to neutralize them [9]. Moreover, RSS can regulate glutathione (GSH) and thioredoxin (Trx-1) systems, activate signaling pathways such as Nrf2-ARE and Sirtuin, and upregulate the expression of antioxidant enzymes [10]. Through these mechanisms, RSS enhances cellular antioxidant capacity, contributing to a robust defense against oxidative stress.

During physical activity, the potential for oxidative stress rises as the body’s metabolic rate increases, leading to higher oxygen consumption and elevated production of ROS [4]. Numerous previous studies have indicated that regular aerobic exercise enhances endogenous antioxidant defenses through various mechanisms. These include increasing plasma antioxidant markers, reducing levels of oxidative markers [11], enhancing endogenous antioxidant enzyme activity [12], activating the nuclear factor erythroid 2–related factor 2 (NRF2) pathway [13], and upregulating oxidative damage repair systems [14]. However, vigorous exercise can elevate oxidative stress levels, which may impair muscle contraction, resulting in muscle fatigue and reduced exercise performance [15]. To relieve muscle damage and fatigue and improve athletic performance, the intake of nutritional supplements with antioxidant properties, either through diet or dietary supplements, is considered an effective approach [16].

Polyphenols, a broad category of nutrients abundant in plants, have been reported to potentially exert indirect antioxidant effects by stimulating the expression of endogenous antioxidant genes [17], regulating enzymes involved in ROS/RNS production [18], and modulating redox-related transcription factors [19]. These mechanisms contribute to reducing oxidative damage during exercise [20], and polyphenols are considered to have potential benefits for exercise performance [21,22].

Black ginger (*Kaempferia parviflora*, KP) is a ginger family species native to Thailand, commonly used in traditional local medicine to treat various conditions, including inflammation, fatigue, and colic [22,23]. KP extract (KPE) extracted from KP rhizomes contains several flavonoids, polymethoxyflavonoids (PMFs), which are natural polyphenols. PMFs in KPE have been reported to have potential antioxidant effects [24]. In vitro, KPE reduced the amount of induced ROS release in cell culture medium [25] and showed dose-dependent scavenging activity against ABTS and DPPH free radicals [26]. Four weeks of KPE administration decreased vascular superoxide production and enhanced NO bioavailability in diabetic rat models [27]. Eight weeks of KPE administration reduced serum malondialdehyde (MDA) levels and boosted the activities of antioxidant enzymes SOD, CAT, and GPx in healthy elderly individuals [28]. In our previous studies on acute KPE administration, we found that KPE may enhance endurance in mice by improving energy metabolism and substrate utilization in both skeletal muscle and liver [22]. However, the effects of long-term KPE administration and long-term exercise intervention on exercise performance have not been fully studied, and there are limited reports on the antioxidant effects of KPE, most of which are in vitro studies [25,26,29], and antioxidant studies on healthy mice in animal model experiments have not been reported. In addition, most in vitro models (such as cell culture) cannot fully simulate complex in vivo systems [30]. Therefore, it is necessary to study whether KPE still has antioxidant effects in the in vivo environment and whether long-term administration of KPE and long-term exercise intervention will affect the endurance performance and antioxidant effects of mice. This study aims to determine whether long-term administration of KPE and long-term exercise intervention can affect the endurance performance and antioxidant capacity of mice.

## 2. Materials and Methods

### 2.1. Animals

Eight-week-old male C57BL/6J mice were obtained from the Takasugi Experimental Animal Supply (Kasukabe, Japan). The mice were acclimatized to laboratory conditions for one week before the initiation of the experiment. Each animal was housed individually in a cage (225 mm (w) × 335 mm (d) × 215 mm (h)) under controlled environmental conditions with a 12-h light-dark cycle (lights on at 08:00 and off at 20:00). All experimental protocols adhered to the Guidelines for Animal Care and Use as set by the Academic Research Ethics Review Committee of Waseda University and received approval under protocol number A22-118.

### 2.2. Preparation of Kaempferia parviflora Extract (KPE)

*Kaempferia parviflora* extract (KPE) was provided by Maruzen Pharmaceuticals Co., Ltd. (Hiroshima, Japan). In brief, dried *Kaempferia parviflora* rhizome was extracted with hydrous ethanol. After extraction, concentration was carried out under reduced pressure and spray-dried with γ-cyclodextrin and dextrin. The components of KPE were quantified through high-performance liquid chromatography. HPLC was carried out using an analytical C18 reversed-phase column (Ascentis Express C18 2.7 µm, 4.6 × 100 mm, Supelco, Bellefonte, PA, USA) and a UV detector (detection wavelengths: 265 nm, Prominence LC system, Shimadzu, Kyoto, Japan) with the column temperature set at 40 °C. The mobile phase consisted of a methanol/H_2_O (55/45) solution containing 0.1% TFA. 5,7,3′,4′-tetramethoxyflavone, 3,5,7,3′,4′-pentamethoxyflavone, 5,7-dimethoxyflavone, 5,7,4′-trimethoxyflavone, 3,5,7-trimethoxyflavone, and 3,5,7,4′-tetramethoxyflavone were used for the standards. The control administration served as the control diet (CE-2, Oriental Yeast, Tokyo, Japan), and the KPE administration was fed a diet containing 3% (*w*/*w*) KPE.

### 2.3. Submaximal Endurance Exercise Capacity Testing Protocol

All the mice were accustomed to a motorized treadmill (Melquest Co., Ltd., Toyama, Japan) by running at 15 m/min for 10 min one week before the first submaximal endurance exercise capacity testing. Before voluntary wheel-running and KPE administration began, all the animals were given normal feed.

The exercise protocol was using a treadmill, according to a previously described method [31,32]. One week before the start of the voluntary wheel-running and KPE administration, all mice were habituated to running on a motorized treadmill, as outlined in earlier studies [22,31]. After this familiarization period, the mice underwent three graded exercise performance tests at distinct time points: the first test was conducted 7 days before the initiation of wheel running, the second took place 1 day after 4 weeks of wheel running, and the third occurred 1 day after 8 weeks of wheel running. The test began with a speed of 9 m/min for 9 min, then increased to 10 m/min, with further increments of 2.5 m/min every 3 min until reaching 25 m/min. The incline started at 0° and was raised by 5° every 9 min, reaching a maximum incline of 15°. Exhaustion was defined as the inability to continue regular treadmill running despite the stimulation of repeated tapping on the back of the mouse. The time of exhaustive running was recorded. After the exercise performance tests, the mice were returned to their cages and continued to be fed.

### 2.4. Voluntary Wheel-Running and KPE Administration

Seven days after the first maximal exercise capacity test, all mice were equally divided into the following four groups based on body weight and exercise ability: C group (control administration + sedentary group, *n* = 8), E group (control administration + voluntary wheel-running group, *n* = 8), K group (KPE administration + sedentary group, *n* = 8), and KE group (KPE administration + voluntary wheel-running group, *n* = 8). The sample size (*n* = 8) was determined by a preliminary test and our previous studies [22,31].

The C and K groups were assigned to individual KN-600U cages ((225 mm (w) × 335 mm (d) × 215 mm (h)) manufactured by (Melquest Co., Ltd., Toyama, Japan). E and KE groups were assigned to KN-600U cages with an activity wheel (0.45 m circumference) for 8 weeks. Wheels were located within the cages and were always freely accessible. Throughout the study, mice were weighed during weekly bedding changes (10:00–11:00). We monitored voluntary wheel-running every 60 s throughout the 8-week experiment using a computer equipped with Actmaster4APC software version1.1.3 (Melquest Co., Ltd., Toyama, Japan) to assess daily average running distance.

### 2.5. Measurement of Plasma Biochemical Parameters and Biomarker of Oxidative Stress Level and Antioxidant Capacity

One week after the third exercise performance test, and following a 4-h fasting period, mice were sacrificed under light anesthesia induced by isoflurane inhalation (Abbott, Tokyo, Japan). Blood samples were collected from the abdominal aorta using heparin, and the soleus and gastrocnemius muscles were rapidly excised and immediately frozen in liquid nitrogen. Plasma was obtained by centrifuging the blood samples at 1500× *g* for 10 min at 4 °C. All collected samples were stored at −80 °C until further analysis. Plasma concentrations of triglyceride (TG), glucose, total cholesterol (TC), low-density lipoprotein cholesterol (LDL), high-density lipoprotein cholesterol (HDL), uric acid, blood urea nitrogen (BUN), creatinine (Cr), aspartate transaminase (AST), alanine transaminase (ALT), albumin, lactate dehydrogenase (LDH), creatine kinase (CK), amylase, and lipase were measured by Kotobiken Medical Laboratories (Tokyo, Japan). The marker of oxidative stress level and antioxidant capacity in plasma was assessed using a Free Radical Elective Evaluator (FREE) and OXY-Adsorbent Test kit, BAP Test kit, and d-ROMs Test kit (Wismerll Co., Ltd., Tokyo, Japan).

The OXY test (colorimetric plasma antioxidant protection assay) evaluates serum’s ability to neutralize oxidative stress induced by hypochlorous acid (HClO) [33]. Unreacted HClO radicals react with a chromogenic solution of N, N-diethyl-p-phenylenediamine, forming a colored complex measured at 505 nm. Results are expressed in μmol/mL. The biological antioxidant potential (BAP) was measured using a BAP kit. This assay evaluates the reduction of Fe^3+^ to Fe^2+^ by assessing the decrease in absorbance of a colored thiocyanate-derived substrate at 505 nm [33]. Results are expressed as μEq of reduced iron ions per. Reactive oxygen metabolites (ROM) were measured using the d-ROMs test kit. This method assesses the concentration of hydroperoxides in the sample [33]. In an acidic buffer (pH 4.8), iron ions catalyze the conversion of hydroperoxides into alkoxyl and peroxyl radicals, which then react with N, N-diethyl-p-phenylenediamine to form a red cation. The absorbance is measured at 505 nm, and results are expressed in CARR U (Carratelli units), with each CARR U corresponding to 0.08 mg H_2_O_2_/100 mL sample.

### 2.6. Real-Time Quantitative Polymerase Chain Reaction (PCR)

Total RNA was isolated from gastrocnemius and soleus muscles using TRI-zol™ Reagent (Thermo Fisher Scientific Inc., Waltham, MA, USA) according to the manufacturer’s protocols. RNA concentration and purity were evaluated with a NanoDrop spectrophotometer (NanoDrop Technologies, Wilmington, DE, USA). Complementary DNA (cDNA) was synthesized from total RNA using the High-Capacity cDNA Reverse Transcription Kit (Applied Biosystems, Foster City, CA, USA). Quantitative real-time PCR (qPCR) was conducted using the Fast 7500 real-time PCR system (Applied Biosystems, Foster City, CA, USA) with Fast SYBR^®^ Green PCR Master Mix (Applied Biosystems, Foster City, CA, USA). The qPCR program included an initial denaturation step at 95 °C for 10 min, followed by 40 cycles at 95 °C for 3 s and annealing at 60 °C for 15 s. The 18S rRNA was used as a reference gene, and gene expression levels were quantified using the ΔΔCT method and normalized to the control group. Data are presented as fold changes relative to the control group. PCR primer pairs for each studied gene are shown in Table 1.

### 2.7. Statistical Analysis

The data are expressed as means ± standard deviation (SD). To evaluate the main effects of KPE administration and/or exercise, a two-way analysis of variance (ANOVA) was applied. Statistical analysis was performed using GraphPad Prism version 9.0 (GraphPad Software, La Jolla, CA, USA). When significant interactions were detected, simple effects analysis followed by Tukey’s post hoc test was used to assess differences between groups. If no significant interaction was found, the main effects were analyzed, and Tukey’s post hoc test was employed to further explore group differences. Effect size analysis in ANOVA was performed by Eta Squared (η^2^). According to the definition of Cohen (1988), the Eta Squared standard is as follows: η^2^ ≈ 0.01: Small effect size: The effect size is small, but has practical significance; η^2^ ≈ 0.06: Medium effect size: The effect size has a more obvious impact; η^2^ ≥ 0.14: Large effect size: The effect is significant and has a strong impact. Furthermore, we analyzed the associations between variables using Pearson’s correlation coefficient. The relationship between the r, R^2^ value and the effect strength is proposed: 0.1 < r < 0.3: small effect, weak correlation; 0.3 < r < 0.5: medium effect, moderate correlation; r > 0.5: large effect, strong correlation. 0 < R^2^ < 1: it means that the independent variable partially explains the variation of the dependent variable. The larger the R^2^, the stronger the explanatory power. Statistical significance was defined as *p* < 0.05.

## 3. Results

### 3.1. Standardized Crude Extract of KPE

The total PMF content of KPE was 9.6%. The composition ratios of 5,7,3′,4′-tetramethoxyflavone, 3,5,7,3′,4′-pentamethoxyflavone, 5,7-dimethoxyflavone, 5,7,4′-trimethoxyflavone, 3,5,7-trimethoxyflavone, and 3,5,7,4′-tetramethoxyflavone were 3%, 26%, 27%, 26%, 5%, and 13%, respectively.

### 3.2. Effect of Long-Term KPE Administration and Voluntary Wheel-Running Training on Mice Submaximal Endurance Exercise Capacity and Total Daily Voluntary Wheel-Running Distance

**Figure 1:**
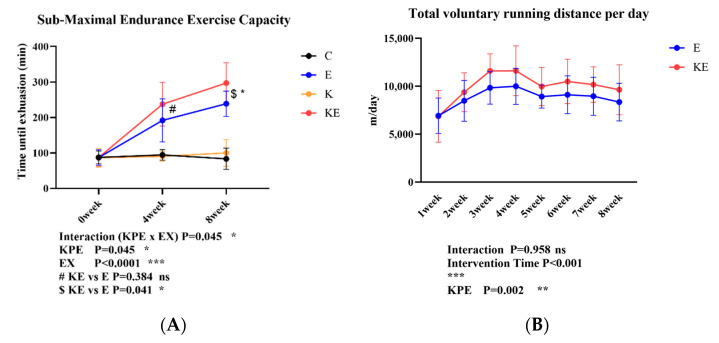
(**A**): The submaximal endurance exercise capacity in three time points. (**B**): The total voluntary wheel-running distance per day in eight time points. C group (control diet + sedentary group; *n* = 8); E group (control diet + voluntary wheel-running group, *n* = 8); K group (KPE administration + sedentary group, *n* = 8); KE group (KPE administration + voluntary wheel-running group, *n* = 8). KPE: a main effect of *Kaempferia parviflora* administration; EX: a main effect of voluntary wheel-running; Interaction: interactive effect between KPE administration and voluntary wheel-running; #: Post hoc comparison between 4 weeks group E and KE; $: Post hoc comparison between 8 weeks group E and KE. Values are means ± standard deviation (SD); ns: no significance observed; * *p* < 0.05, ** *p* < 0.01, *** *p* < 0.001.

We evaluated the effects of KPE administration and voluntary wheel-running training on the mice’s submaximal endurance exercise capacity and workload at 0 weeks, 4 weeks, and 8 weeks. As shown in Figure 1A, after 4 weeks and 8 weeks of voluntary wheel-running training, submaximal endurance exercise capacity was significantly enhanced in both the E and KE groups compared with the sedentary group (C and K) (*p* < 0.001, η^2^ = 0.69). Compared with group E, the submaximal endurance exercise capacity of group KE was significantly enhanced after 8 weeks of KPE administration ($ KE vs. E *p* = 0.041, η^2^ = 0.19). In Figure 1B, starting from the second week of KPE administration, the daily voluntary running distance of the KE group increased significantly compared with the E group (*p* < 0.002, η^2^ = 0.08).

### 3.3. Effect of Long-Term KPE Administration on Mice Plasma Oxidative Stress and Anti-Oxidative Stress Capacity

**Figure 2:**
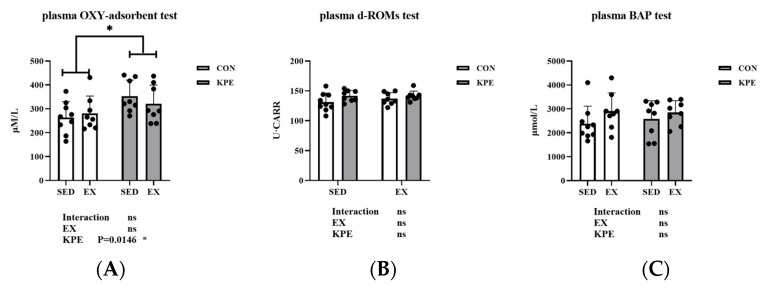
(**A**): The colorimetric determination of plasmatic oxidation protection test in mice plasma. (**B**): The reactive oxygen metabolites test in mice plasma. (**C**): The biological anti-oxidant potential test in mice plasma. SED, sedentary; KPE: *Kaempferia parviflora* extract administration; EX: voluntary wheel-running; Con: control administration; Interaction: interactive effect between KPE administration and voluntary wheel-running; Values are means ± standard deviation (SD); ns: no significance observed; * *p* < 0.05.

As shown in Figure 2, KPE administration significantly enhanced the oxidative protection capacity in mouse plasma (Figure 2A, *p* = 0.0146, η^2^ = 0.19), but had no significant effect on reactive oxygen metabolites (Figure 2B) or biological antioxidant potential (Figure 2C). Additionally, exercise (EX) and the interaction between KPE and exercise did not show significant differences in any of these parameters. These findings suggest that KPE exerts its antioxidant effects primarily by enhancing plasma oxidative protection capacity rather than by altering reactive oxygen metabolites or biological antioxidant potential.

### 3.4. Correlation between Submaximal Endurance Exercise Capacity, Total Daily Voluntary Wheel-Running Distance, and Plasma Antioxidant Capacity

**Figure 3:**
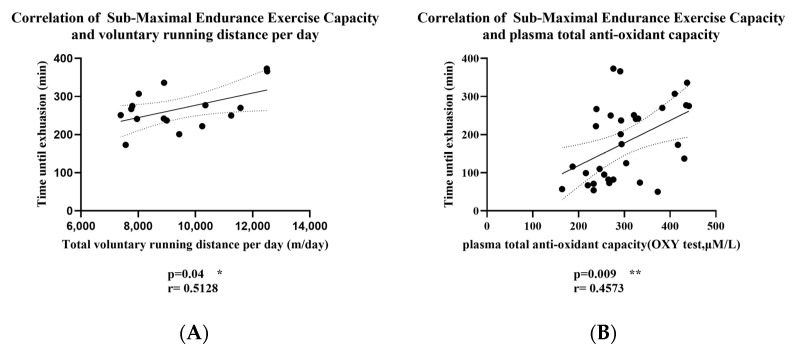
(**A**) Correlation of Submaximal Endurance Exercise Capacity and Voluntary Running Distance per day; (**B**) Correlation of Submaximal Endurance Exercise Capacity and Plasma Total Antioxidant Capacity; The r value indicates the strength and direction of the linear relationship between two variables; solid lines: best-fit line; dashed lines: 95% confidence bands of the best-fit line. * *p* < 0.05, ** *p* < 0.01.

Considering the weekly changes in the voluntary wheel-running distance of mice, we averaged the running distances over the 8 weeks for correlation analysis. From Figure 3, we found a significant positive correlation between submaximal endurance exercise capacity and both voluntary running distance per day and plasma total antioxidant capacity in mice. Specifically, submaximal endurance capacity was moderately correlated with daily voluntary running distance (r = 0.5128, R^2^ = 0.26, *p* = 0.04) and significantly correlated with plasma total antioxidant capacity (r = 0.4573, R^2^ = 0.2, *p* = 0.009). These results suggest that increased voluntary running distance and higher plasma antioxidant capacity may contribute to enhanced endurance performance in mice.

### 3.5. Effect of Long-Term KPE Administration and Voluntary Wheel-Running Training on Metabolism Regulation in Plasma

**Figure 4:**
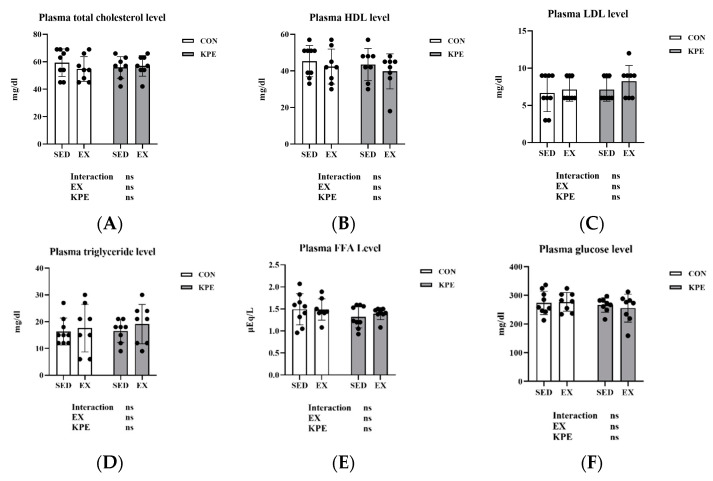
(**A**) plasma total cholesterol; (**B**) plasma HDL; (**C**) plasma LDL; (**D**) plasma triglyceride; (**E**) plasma FFA; and (**F**) plasma glucose concentrations immediately after exhaustion. FFA, free fatty acids; LDL, low-density lipoprotein cholesterol; HDL, high-density lipoprotein cholesterol; SED, sedentary; EX, exercise; Con, control; KPE, *Kaempferia parviflora* extract administration; ns, no significance observed.

In Figure 4, there were no significant changes with long-term KPE administration or voluntary wheel-running training in plasma total cholesterol, HDL, LDL, triglyceride, FFA, or glucose.

### 3.6. Effect of Long-Term KPE Administration and Voluntary Wheel-Running Training on Oxidative Stress Response Regulators Gene Expression in Soleus Muscle

**Figure 5:**
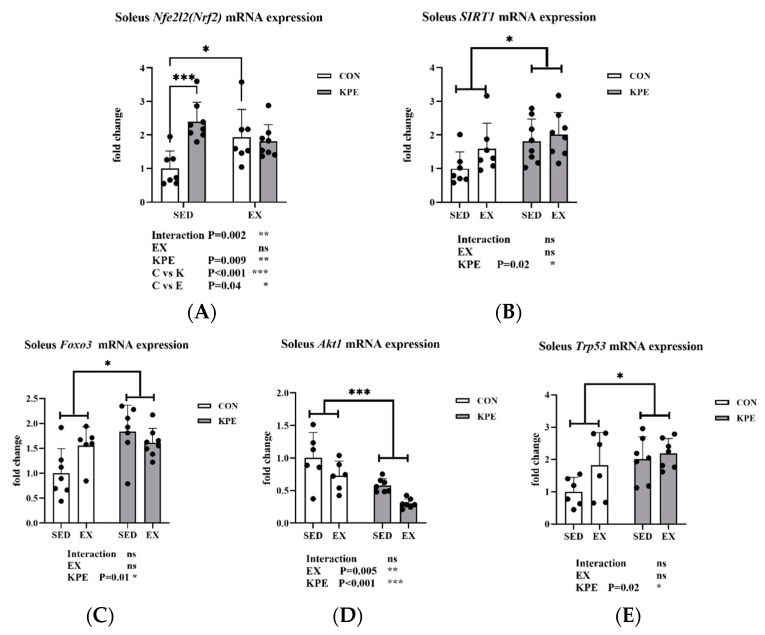
mRNA expression of (**A**) *Nfe212* (*Nrf2*); (**B**) *Sirt1;* (**C**) *Foxo3*; (**D**) *Akt1;* (**E**) *Trp53* in soleus muscle. *Sirt1*, sirtuin 1; *Nfe2l2*(*Nrf2*), nuclear factor, erythroid-derived 2, like 2; *Foxo3*, forkhead box O3; *Trp53*, transformation-related protein 53; *Akt1*, thymoma viral proto-oncogene 1; SED, sedentary; KPE: KPE administration; EX: voluntary wheel-running; Con: control administration; Interaction: interactive effect between *Kaempferia parviflora* extract administration and voluntary wheel-running; ns, no significance observed; * *p* < 0.05, ** *p* < 0.01, *** *p* < 0.001.

Figure 5 showed that long-term KPE administration significantly increased the gene expression of *Nfe212* (*Nrf2*) (*p* = 0.009, η^2^ = 0.24), *Sirt1* (*p* = 0.02, η^2^ = 0.2), *Foxo3* (*p* = 0.01, η^2^ = 0.24), and *Trp53* (*p* = 0.02, η^2^ = 0.23) in the soleus muscle. The gene expression of *Akt1* was significantly decreased by long-term KPE administration (*p <* 0.001, η^2^ = 0.51) and voluntary wheel-running training (*p* = 0.005, η^2^ = 0.3).

### 3.7. Effect of Long-Term KPE Administration and Voluntary Wheel-Running Training on Superoxide Dismutase’s Gene Expression in Soleus Muscle

**Figure 6:**
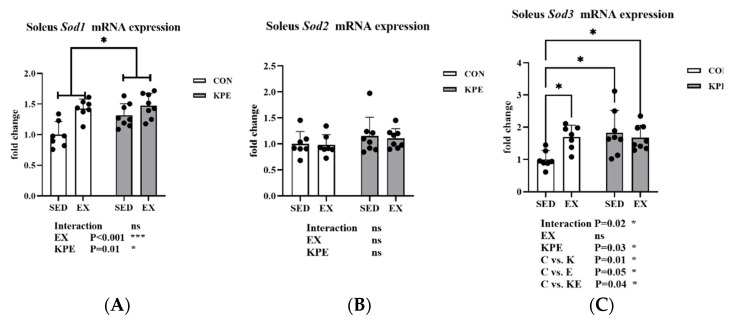
mRNA expression of (**A**) *Sod1*; (**B**) *Sod2*; (**C**) *Sod3* in soleus muscle. *Sod1*, superoxide dismutase 1, soluble; *Sod2*, superoxide dismutase 2, mitochondrial; *Sod3*, superoxide dismutase 3, extracellular; SED, sedentary; KPE: KPE administration; EX: voluntary wheel-running; Con: control administration; Interaction: interactive effect between *Kaempferia parviflora* extract administration and voluntary wheel-running; ns, no significance observed; * *p* < 0.05, *** *p* < 0.001.

As shown in Figure 6, there was a significant increase in *Sod1* gene expression with long-term KPE administration (*p* = 0.01, η^2^ = 0.21) and voluntary wheel-running training (*p* < 0.001, η^2^ = 0.41). Long-term KPE administration significantly increased *Sod3* gene expression in soleus muscle in sedentary groups (C vs. K, *p* = 0.01). Long-term voluntary wheel-running training significantly increased *Sod3* gene expression in the control administration groups (C vs. E, *p* = 0.05) but had no significant effect in the KPE administration groups. There was no significant difference in *Sod2* gene expression.

### 3.8. Effect of Long-Term KPE Administration and Voluntary Wheel-Running Training on Glutathione Metabolism and Antioxidant Defense-Related Gene Expression in Soleus Muscle

**Figure 7:**
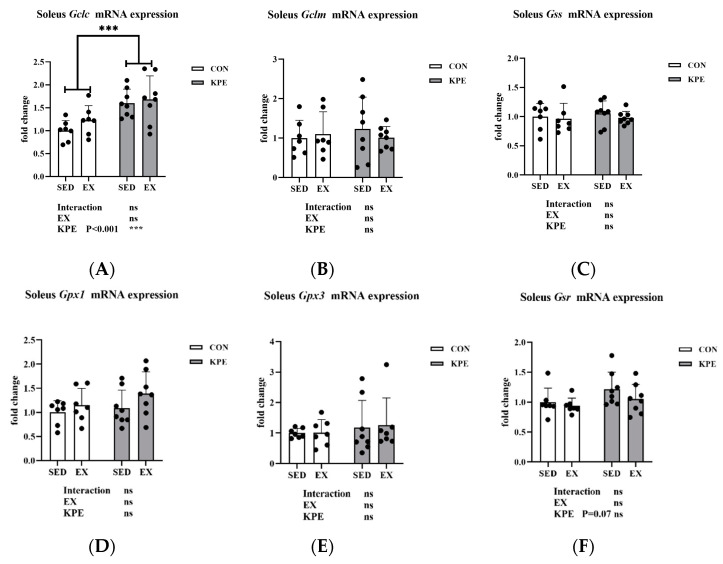
mRNA expression of (**A**) *Gclc*; (**B**) *Gclm*; (**C**) *Gss*; (**D**) *Gpx1*; (**E**) *Gpx3*; and (**F**) *Gsr* in soleus muscle. *Gclc*, glutamate-cysteine ligase, catalytic subunit; *Gclm*, glutamate-cysteine ligase, modifier subunit; *Gss*, glutathione synthetase; *Gpx1*, glutathione peroxidase 1; *Gpx3*, glutathione peroxidase 3; *Gsr*, glutathione reductase; SED, sedentary; KPE: KPE administration; EX: voluntary wheel-running; Con: control administration; Interaction: interactive effect between *Kaempferia parviflora* extract administration and voluntary wheel-running; ns, no significance observed; *** *p* < 0.001.

From Figure 7, the long-term KPE administration significantly increased *Gclc* gene expression (*p* < 0.001, η^2^ = 0.38) and showed a tendency to increase *Gsr* gene expression (*p* = 0.07, η^2^ = 0.12) in the soleus muscle. Neither long-term KPE administration nor voluntary wheel-running training showed a significant effect on the expression of the *Gclm, Gss, Gpx1, and Gpx3* genes.

### 3.9. Effect of Long-Term KPE Administration and Voluntary Wheel-Running Training on Antioxidant-Related Gene Expression in Soleus Muscle

**Figure 8:**
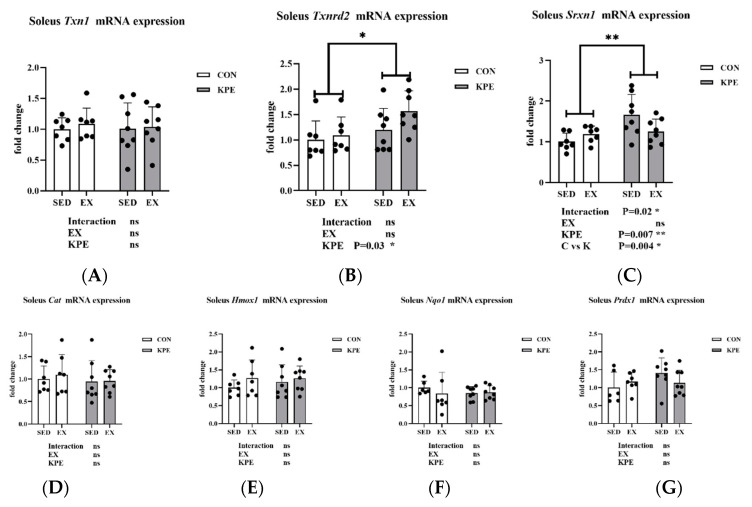
mRNA expression of (**A**) *Txn1;* (**B**) *Txnrd2*; (**C**) *Srxn1*; (**D**) *Cat*; (**E**) *Hmox1*; (**F**) *Nqo1;* and (**G**) *Prdx1* in soleus muscle. *Txn1*, thioredoxin 1; *Txnrd2*, thioredoxin reductase 2; *Srxn1*, sulfiredoxin 1 homolog (*S. cerevisiae*); *Cat*, catalase; *Hmox1*, heme oxygenase 1; *Nqo1* NAD(P)H dehydrogenase, quinone 1; *Prdx1*, peroxiredoxin 1; SED, sedentary; KPE: KPE administration; EX: voluntary wheel-running; Con: control administration; Interaction: interactive effect between *Kaempferia parviflora* extract administration and voluntary wheel-running; ns, no significance observed; * *p* < 0.05, ** *p* < 0.01.

From Figure 8, both *Txnrd2* (*p* = 0.03, η^2^ = 0.17) and *Srxn1* (*p* = 0.007, η^2^ = 0.25) gene expression levels were significantly increased by long-term KPE administration, while *Txn1, Cat, Hmox1, Nqo1*, and *Prdx1* gene expression remained unchanged in soleus muscle. *Srxn1* gene expression also showed a significant interaction effect between long-term KPE administration and sedentary behavior (C vs. K, *p* = 0.004).

### 3.10. Effect of Long-Term KPE Administration and Voluntary Wheel-Running Training on Antioxidant-Related Gene Expression in Gastrocnemius Muscle

**Figure 9:**
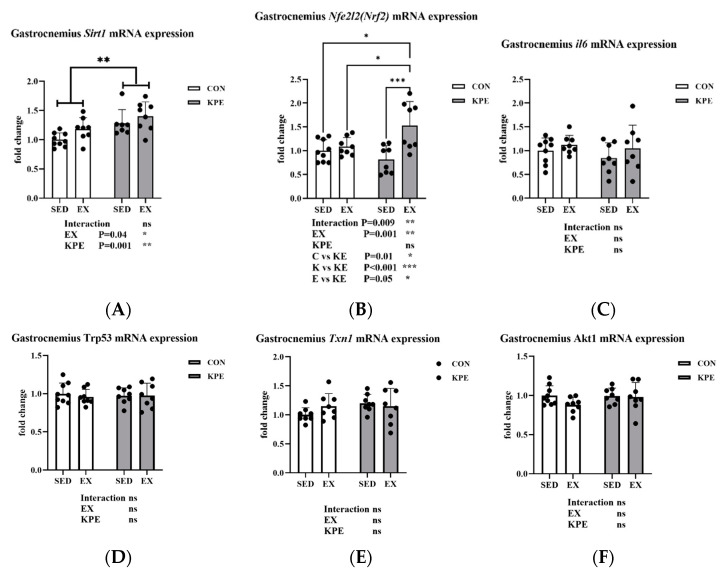
mRNA expression of (**A**) *Sirt1*; (**B**) *Nfe2l2*; (**C**) *Il6*; (**D**) *Trp53*; (**E**) *Txn1*; and (**F**) *Akt1* in gastrocnemius muscle *Sirt1*, sirtuin 1; *Nfe2l2*, nuclear factor, erythroid-derived 2, like 2; *Il6*, interleukin 6; *Trp53*, transformation-related protein 53; *Txn1*, thioredoxin 1; *Akt1*, thymoma viral proto-oncogene 1; SED, sedentary; KPE: KPE administration; EX: voluntary wheel-running; Con: control administration; Interaction: interactive effect between *Kaempferia parviflora* extract administration and voluntary wheel-running; ns, no significance observed; * *p* < 0.05, ** *p* < 0.01, *** *p* < 0.001.

As shown in Figure 9, Long-term KPE administration (*p* = 0.001, η^2^ = 0.31) and voluntary wheel-running training (*p* = 0.04, η^2^ = 0.14) significantly increased *Sirt1* gene expression levels in the gastrocnemius muscle, while *Il6, Trp53, Akt1*, and *Txn1* gene expression levels remained unchanged under these conditions. The gene expression of *Nfe2l2 (Nrf2)* was significantly increased by long-term voluntary wheel-running (*p* = 0.001, η^2^ = 0.31) and the interaction with KPE administration (*p* = 0.009).

## 4. Discussion

This study described the effect of long-term KPE administration and voluntary wheel-running training on the submaximal endurance exercise capacity of mice and the possible mechanisms. In contrast to forced treadmill exercise, running in a voluntary wheel more closely resembles the natural running behavior of mice [34,35,36], allowing them to exercise in a non-stressful environment without disturbing their circadian rhythm [34]. Therefore, we chose voluntary wheel running as a long-term exercise intervention for mice.

Consistent with previous studies [37], our study found that voluntary wheel-running training significantly enhanced submaximal endurance exercise time in mice. Although there was no difference in exercise time between sedentary groups, in voluntary wheel-running training groups, the exercise time was 24.7% longer in the 8-week KPE supplementation group compared to the control group (E vs. KE, 238.8 ± 35.5 vs. 297.3 ± 57.3) (Figure 1A). Moreover, the daily voluntary running distance in the KPE supplementation group was significantly higher than in the control group (approximately 20% more) (Figure 1B).

Previous research has suggested a correlation between endurance exercise performance and voluntary wheel-running activity in rodents [38]. For example, a study found that mice selectively bred for high spontaneous wheel-running activity exhibited greater endurance exercise capacity, with a significant increase in daily wheel-running activity [39]. Consistent with previous studies, our study found a significant positive correlation between daily running distance and submaximal endurance exercise time in mice (Figure 3A). Therefore, we speculate that compared with the control group, long-term KPE administration increased the daily running distance of mice, further improving submaximal endurance exercise capabilities.

Since acute administration of KPE improved the endurance of mice by enhancing the energy metabolism pathways and substrate utilization in muscle and liver in our previous experiments, we tried to detect the metabolites in plasma in this study [22]. Regarding the plasma biochemical data (Figure 4), neither long-term KPE supplementation nor voluntary wheel running had significant effects on plasma metabolites at rest. This result is consistent with previous research [40]. The lack of significant changes in plasma metabolites at rest may be attributed to the adaptive regulation in response to long-term KPE administration and voluntary wheel-running training.

In our study, we found that long-term intake of KPE significantly increased the plasma antioxidant capacity of mice at rest (Figure 2A) and was positively correlated with submaximal endurance exercise time (Figure 3B). Therefore, we speculate that the effect of long-term intake of KPE on exercise capacity is achieved by affecting the antioxidant level in mice. Since many studies have reported that polyphenols produce antioxidant effects by activating the Nrf2 signaling pathway [19,41,42,43], we decided to investigate changes in the Nrf2 signaling pathway in skeletal muscle, which is closely related to exercise capacity.

The Nrf2 (Nuclear factor erythroid 2-related factor 2)/ARE (Antioxidant Response Element) pathway is a key signaling mechanism for cells to combat oxidative stress [44]. Nrf2, a transcription factor, is released from its inhibitor Keap1 during oxidative stress, translocates to the nucleus, and binds to the ARE, triggering the expression of antioxidant genes [42]. These genes, such as *Sod* and *Gpx*, help protect cells from oxidative damage and maintain redox balance [45]. Supplements containing polyphenols such as quercetin and resveratrol have garnered interest due to their potential to boost endurance performance and strengthen the body’s antioxidant defenses through the activation of the Nfe212 (Nrf2) signaling pathway [4]. Similar to previous studies, our experiment found that long-term KPE administration combined with voluntary wheel-running training significantly increased the mRNA expression levels of *Nfe212* (*Nrf2*) and *Sirt1* in the gastrocnemius muscle of mice (Figure 9). In the soleus muscle, long-term KPE administration significantly increased the gene expression levels of *Nfe212* (*Nrf2*), *Sirt1*, *Akt1*, *Foxo3*, and *Trp53*, and voluntary wheel-running training significantly reduced the gene expression of *Akt1* (Figure 5).

*Sirt1* is a NAD+-dependent deacetylase and a member of the sirtuin protein family, playing a crucial role in regulating metabolism, aging, inflammation, and oxidative stress [46]. *Sirt1* has been reported to directly increase the expression of antioxidant genes, including *Sod2*, *hmox1*, and *Nqo-1* [47]. Therefore, KPE intake may enhance cellular antioxidant capacity by upregulating the expression of *Nfe212* (*Nrf2*) and *Sirt1*, thereby activating the Nrf2/ARE pathway.

The Foxo3 transcription factor can be deacetylated and activated by *Sirt1*. Once activated, *Foxo3* further promotes the expression of antioxidant genes (*Sod2*, *Cat*, *Gpx*) [47], enhancing the cell’s tolerance to oxidative stress [48]. However, Akt1 can phosphorylate Foxo3, causing its translocation from the nucleus to the cytoplasm, thereby inhibiting Foxo3’s transcriptional activity [49]. This suggests that under certain conditions, Akt1 may reduce the cellular response to oxidative stress. Therefore, long-term KPE administration may enhance cellular antioxidant capacity by reducing the gene expression of *Akt1* while increasing the gene expression of *Foxo3* and *Sirt1*.

Furthermore, Trp53, a critical tumor suppressor gene, plays an important role in responding to DNA damage and oxidative stress [50]. Studies have shown that low levels of oxidative stress can activate Trp53, triggering antioxidant responses and delaying aging, while high levels of oxidative stress can activate Trp53’s pro-oxidant targets, accelerating oxidative stress and apoptosis to prevent cancer [51]. In this study, KPE supplementation led to an increase in *Trp53* expression in the soleus muscle, which may reflect the positive regulatory effects of long-term KPE administration under low oxidative stress conditions.

In summary, the enhancement of antioxidant capacity in skeletal muscle at rest by long-term KPE intake may be achieved through the activation of the Nrf2/ARE and *Sirt1*/*Foxo3* pathways, along with the regulation of *Akt1* and *Trp53* activity. The adaptive response induced by voluntary wheel-running training is mainly reflected through the Nrf2/ARE pathway and regulating the activity of *Akt1*.

Given that long-term KPE administration has a more pronounced effect on the soleus muscle and that extended exercise, such as voluntary wheel running, predominantly affects the soleus muscle due to its high concentration of type 1 muscle fibers [22], we focused our analysis on the expression of antioxidant-related genes in the soleus muscle.

Sod1, Sod2, and Sod3 are three isoforms of superoxide dismutase, responsible for clearing superoxide anions (O_2_^•−^) inside and outside cells and preventing damage caused by oxidative stress [45]. Sod2 acts mainly in mitochondria, *Sod1* acts in the cytoplasm and mitochondrial space, and Sod3 protects cells in the extracellular matrix and blood [52]. Their expression is regulated by the Nfe212 (Nrf2) signaling pathway [53]. In this study, long-term KPE administration and voluntary wheel-running training significantly increased the expression of *Sod1* and *Sod3* in the soleus muscle, while the expression of *Sod2* did not change (Figure 6). This may indicate that long-term KPE administration and voluntary wheel-running training enhance the antioxidant capacity inside and outside cells in the resting state.

Glutathione (GSH) is the predominant antioxidant found in aerobic cells, serving a crucial role in shielding cells from oxidative stress by scavenging free radicals and inhibiting lipid peroxidation [54]. Gclc is the key rate-limiting enzyme for glutathione synthesis, while Gsr is responsible for reducing oxidized glutathione to reduced GSH, thereby maintaining intracellular antioxidant capacity [55]. *Nfe212* (*Nrf2*) regulates the expression of *Gclc* and *Gsr* by activating the antioxidant response element (ARE) and enhancing the cellular antioxidant defense mechanism [56,57]. In our study, long-term KPE administration significantly increased the gene expression of *Gclc*, and the expression of *Gsr* also showed an upward trend (Figure 7), which indicates that long-term KPE administration may increase the synthesis and reduction rate of GSH in the resting state of skeletal muscle, while voluntary wheel-running training has no obvious effect on this pathway.

*Txnrd2* and *Srxn1* are endogenous antioxidant enzymes that play roles in cellular antioxidant defense through different pathways [13]. Txnrd2 mainly catalyzes the reduction reaction of thioredoxin (Trx) in mitochondria and maintains mitochondrial redox balance [58]. Srxn1 enhances cellular antioxidant capacity by repairing oxidatively damaged proteins, and *Nfe212* (*Nrf2*) can activate the expression of *Srxn1* under oxidative stress conditions [59]. In our study, long-term KPE administration significantly increased the expression of *Txnrd2* and *Srxn1* to enhance cellular antioxidant defense by maintaining mitochondrial function and repairing oxidative damage (Figure 8).

In summary, the long-term KPE administration may exert its antioxidant effects in skeletal muscle primarily by activating the Nrf2-ARE pathway, leading to the transcription of downstream antioxidant genes such as *Sod1*, *Sod3*, *Gclc*, *Gsr*, *Txnrd2*, and *Srxn1*, while voluntary wheel exercise also activates some related pathways.

Although KPE also activates the Sirt1/Foxo3 pathway, downstream genes such as *Sod2*, *Cat*, *Gpx1*, *Hmox1*, and *Nqo1* are not activated, which is consistent with the research that polyphenols mostly produce antioxidant effects by activating the Nfe212 (Nrf2) signaling pathway [19,20,42,43]. We speculate that KPE’s antioxidant effects may enhance skeletal muscle antioxidant capacity via the Nrf2-ARE pathway and increase plasma antioxidant levels, thereby improving submaximal endurance in mice.

The limitation of this study is that blood biochemistry and muscle samples were only collected when the mice were at rest and were not collected immediately after exercise. The role of long-term KPE administration in the exercise state of mice needs further study. In terms of statistical analysis, we followed the analysis of Pearson’s correlation coefficient of previous studies, but the Spearman correlation coefficient may be more appropriate according to the characteristics of the variables. According to relevant literature, we found that the induction pathway of RSS to antioxidant factors overlaps with the pathway in our conclusion, which suggests that KPE administration may have a potential effect on the level of RSS. Unfortunately, due to insufficient samples, we were unable to measure the levels of other oxidation-related products, such as RSS in plasma and muscle, which needs to be carried out in subsequent studies.

## 5. Conclusions

**Figure 10:**
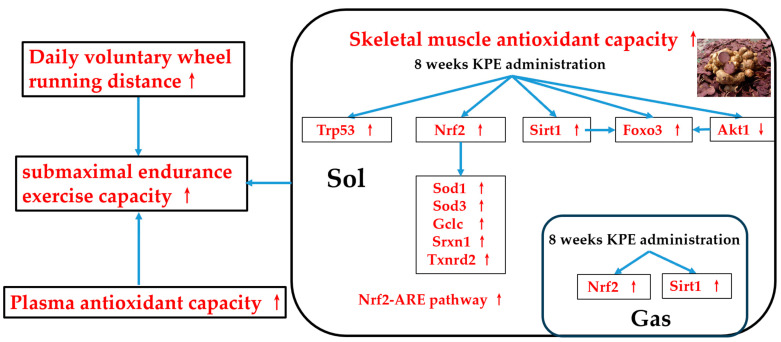
The suggested mechanisms of KPE administration enhancing submaximal endurance exercise capacity. *Sirt1*, sirtuin 1; *Nfe2l2 (nrf2)*, nuclear factor, erythroid-derived 2, like 2; *Foxo3*, forkhead box O3; *Trp53*, transformation-related protein 53; *Akt1*, thymoma viral proto-oncogene 1; *Sod*, superoxide dismutase family; *Gclc*, glutamate-cysteine ligase, catalytic subunit; *Txnrd2*, thioredoxin reductase 2; *Srxn1*, sulfiredoxin 1 homolog (*S. cerevisiae*); GAS, gastrocnemius muscle; SOL, soleus muscle. Arrows, Potential effects of KPE administration.

This study found that long-term KPE administration can enhance the submaximal endurance exercise capacity of mice. The possible mechanism of endurance enhancement is to activate the antioxidant pathway centered on the Nrf2-ARE pathway in skeletal muscle to improve the antioxidant capacity of skeletal muscle and plasma and increase the daily running distance of mice, which ultimately manifests as improving the submaximal endurance exercise capacity of mice (Figure 10).

## Figures and Tables

**Table 1:** Primer sequence for real-time PCR analysis.

Gene Symbol for Primer	Forward	Reverse
*Rn18s* (*NR_003278.3*)	TTCTGGCCAACGGTCTAGACAAC	CCAGTGGTCTTGGTGTGCTGA6
*Sirt1* (*NM_001159589.2*)	ACGGTATCTATGCTCGCCTTGC	GACACAGAGACGGCTGGAACTG
*Nfe212* (*Nrf2*); *NM_001399226.1*	AGTTGCCACCGCCAGGACTA	CGTGCTCAGAAACCTCCTTCCAAA
*Foxo3* (*NM_001376967.1*)	TCCGTGAGCAAGCCGTGTACT	AGCAGGTCGTCCATGAGGTTCT
*Trp53* (*NM_001127233.2*)	GCATGAACCGCCGACCTATCCT	CAGGGCAGGCACAAACACGAA
*Akt1* (*NM_001165894.2*)	AAGGAGGTCATCGTCGCCAAGG	CGGTCGTGGGTCTGGAATGAGT
*Sod1* (*NM_011434.2*)	GAACCAGTTGTGTTGTCAG	GTACAGCCTTGTGTATTGTC
*Sod2* (*NM_013671.3*)	CAACTCAGGTCGCTCTTC	TGATAGCCTCCAGCAACT
*Sod3* (*NM_011435.3*)	CTTGTTCTACGGCTTGCTA	CTATCTTCTCAACCAGGTCAA
*Gclm* (*NM_008129.4*)	CATGGCTTCGCCTCCGATTGA	GCTGCTCCAACTGTGTCTTGTC
*Gss* (*NM_001291111.1*)	GCTGTGGTGTACTTCCGAGATGG	GGACACTTGGCAGCACGAGAT
*Gpx1* (*NM_001329527.1*)	GCAATCAGTTCGGACACCAGAAT	CTCACCATTCACTTCGCACTTCTC
*Gpx3* (*NM_001329860.1*)	ATGGCGGTATGAGTGGTA	CAAGGTATTGGTCTGTCAGA
*Gclc* (*NM_010295.2*)	CACATCTACCACGCAGTCAAGGA	AGTCTCAAGAACATCGCCTCCATT
*Gsr* (*NM_010344.4*)	CGGCGTGGAGGTGTTGAAGTT	ACATCTGGAATCATGGTCGTGGTG
*Txn1* (*NM_011660.3*)	GCTTGTCGTGGTGGACTTCTCTG	CAGCAACATCCTGGCAGTCATCC
*Cat* (*NM_080483.3*)	GATGGAGAGGCAGTCTATTG	ATTGGCGATGGCATTGAA
*Hmox1* (*NM_010442.2*)	GACCGCCTTCCTGCTCAACATT	CCTCTGACGAAGTGACGCCATC
*Nqo1* (*NM_008706.5*)	GGTAGCGGCTCCATGTACTCTC	ACGCAGGATGCCACTCTGAATC
*Prdx1* (*NM_011034.5*)	GCCGCTCTGTGGATGAGATTATAC	GCTTGATGGTATCACTGCCAGGTT
*Txnrd2* (*NM_001353143.1*)	GCACAGGTGATGCAGACAGTAGG	TAGCCTCAGCAACCAGTCACAGTA
*Srxn1* (*NM_029688.6*)	CCCAGGGTGGCGACTACTACTA	GCTTGGCAGGAATGGTCTCTCT
*Il6* (*NM_001314054.1*)	CTTGGGACTGATGCTGGTGACA	GCCTCCGACTTGTGAAGTGGTA

*Rn18s*, 18 s ribosomal RNA; *Sirt1*, sirtuin 1; *Nfe2l2*, *nuclear factor*, erythroid-derived 2, like 2; *Foxo3*, forkhead box O3; *Trp53*, transformation-related protein 53; *Akt1*, thymoma viral proto-oncogene 1; *Sod1*, superoxide dismutase 1, soluble; *Sod2*, superoxide dismutase 2, mitochondrial; *Sod3*, superoxide dismutase 3, extracellular; *Gclm*, glutamate-cysteine ligase, modifier subunit; *Gss*, glutathione synthetase; *Gpx1*, glutathione peroxidase 1; *Gpx3*, glutathione peroxidase 3; *Gclc*, glutamate-cysteine ligase, catalytic subunit; *Gsr*, glutathione reductase; *Txn1*, thioredoxin 1; *Cat*, catalase; *Hmox1*, heme oxygenase 1; *Nqo1* NAD(P)H dehydrogenase, quinone 1; *Prdx1*, peroxiredoxin 1; *Txnrd2*, thioredoxin reductase 2; *Srxn1*, sulfiredoxin 1 homolog (*S. cerevisiae*); *Il6*, interleukin 6.

## Data Availability

Data is contained within the article.
